# Monitoring within-farm transmission dynamics of antimicrobial-resistant *Campylobacter* in dairy cattle using broth microdilution and long-read whole genome sequencing

**DOI:** 10.1038/s41598-023-39588-3

**Published:** 2023-08-02

**Authors:** Medelin Ocejo, Beatriz Oporto, José Luis Lavín, Ana Hurtado

**Affiliations:** 1 Animal Health Department, NEIKER – Basque Institute for Agricultural Research and Development, Basque Research and Technology Alliance (BRTA), Bizkaia Science and Technology Park 812L, 48160 Derio, Bizkaia Spain; 2 Applied Mathematics Department, NEIKER – Basque Institute for Agricultural Research and Development, Basque Research and Technology Alliance (BRTA), Bizkaia Science and Technology Park 812L, 48160 Derio, Bizkaia Spain

**Keywords:** Computational biology and bioinformatics, Microbiology, Molecular biology

## Abstract

*Campylobacter jejuni* and *Campylobacter coli* are important foodborne zoonotic pathogens and cause for concern due to the increasing trend in antimicrobial resistance. A long-run surveillance study was conducted in animals from different age groups in five dairy cattle farms to investigate the within-farm diversity and transmission dynamics of resistant *Campylobacter* throughout time. The resistance phenotype of the circulating isolates (170 *C. jejuni* and 37 *C. coli*) was determined by broth microdilution and a selection of 56 isolates were whole genome sequenced using the Oxford-Nanopore long-fragment sequencing technology resulting in completely resolved and circularized genomes (both chromosomes and plasmids). *C. jejuni* was isolated from all farms while *C. coli* was isolated from only two farms, but resistance rates were higher in *C. coli* than in *C. jejuni* and in calves than in adult animals. Some genotypes (e.g. ST-48, *gyrA*_T86I/*tet*(O)/*bla*_OXA-61_ in farm F1; ST-12000, *aadE*-Cc/*tet*(O)/*bla*_OXA-489_ in F4) persisted throughout the study while others were only sporadically detected. Acquisition of extracellular genes from other isolates and intracellular mutational events were identified as the processes that led to the emergence of the resistant genotypes that spread within the herds. Monitoring with Oxford Nanopore Technologies sequencing helped to decipher the complex molecular epidemiology underlying the within-farm dissemination of resistant* Campylobacter.*

## Introduction

Campylobacteriosis is the main cause of human bacterial gastroenteritis in industrialized countries with the species *Campylobacter jejuni* and *Campylobacter coli* being the primary contributors. After poultry, cattle are considered a major source of *Campylobacter* transmission to humans through the consumption of contaminated food and/or water or by direct contact with the animals or their faeces^1^. Since *Campylobacter* infections are usually self-limited, antimicrobial therapy is only indicated in systemic and severe infections. Nevertheless, antimicrobial-resistant campylobacters are a matter of concern because they put infection treatment options at risk. Fluoroquinolones (FQs) and tetracyclines, often prescribed as empirical therapy for diarrhea^2^, have reduced efficacy due to the high levels of resistance. Macrolides have therefore become the antimicrobials of choice for laboratory-confirmed cases of severe campylobacteriosis^3,4^.

Our previous findings based on cross-sectional epidemiological studies carried out in the Basque Country (northern Spain) showed that dairy cattle represent an important reservoir for resistant *Campylobacter*^5,6^ and that the prevalence of resistance to medically critically important antimicrobials, such as FQs, is increasing whereas resistance against macrolides remains low^6,7^. Acquisition of certain antimicrobial resistance traits has been associated with increased bacterial fitness (e.g*.* the C257T point mutation in the *gyrA* gene)^8,9^ whereas others lead to fitness decrease (e.g*.* the point mutation 2075G in the 23S rRNA gene)^10,11^. To some extent, this might explain the currently observed high rates of FQ resistance and the low rates of macrolide resistance in *Campylobacter* spp. from livestock^12^.

Several studies have reported variability in the faecal shedding patterns of *Campylobacter* associated with age and differences in the within-farm persistence of distinct genotypes ^13–15^. However, the within-herd transmission dynamics of resistant genotypes have not been fully elucidated, and gaps still exist regarding the spread of antibiotic resistance. Considering the high correlation between resistance inference based on phenotype using minimum inhibitory concentrations (MIC) and genotype using whole genome sequencing (WGS) in *Campylobacter*^16^ and the power of WGS for the in-depth genomic characterization of bacteria^17^, here, a longitudinal study was carried out in five dairy cattle farms for a period of 16 months to investigate whether certain resistant genotypes could be more adapted to long-term herd colonization. Phenotypic antimicrobial susceptibility testing and WGS were combined to characterize the isolates recovered from animals of different age groups (calves, heifers, and lactating cows) collected over time. To assure complete assemblies of circular bacterial genomes and plasmids and obtain accurate information about the location of the antimicrobial resistance genes (ARGs), long-read sequencing based on the Oxford Nanopore technology (ONT) was used. Understanding the complex epidemiology and dynamics of the acquisition and spread of antibiotic resistance in *C. jejuni* and *C. coli* within dairy cattle farms would aid in setting baselines for recommendations for on-farm management practices.

## Results

### Antimicrobial resistance profiles distribution

Presence of *Campylobacter* spp., as determined by real-time PCR analysis of a loopful of bacterial growth on CASA® plates, was detected in 98.6% of the samples, 88.8% being PCR-positive to *C. jejuni*, *C. coli* or both. Whereas *C. jejuni* was isolated in all farms, *C. coli* was only found in two of them, i.e., in F4, where *C. coli* was the predominant species in all age groups, and in F5, where *C. coli* was only isolated from calves while *C. jejuni* was found in all age groups.

**Figure 1:**
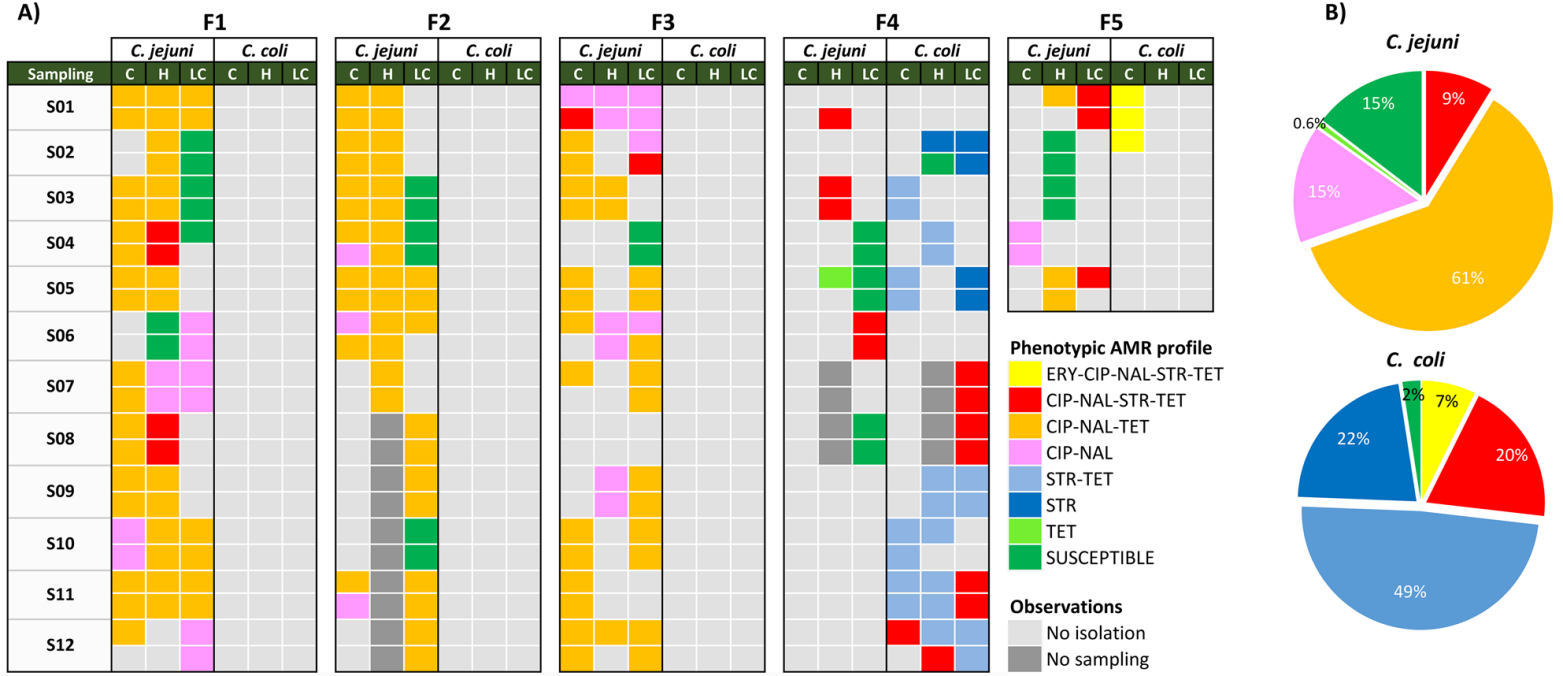
(**A**) Distribution of phenotypic antimicrobial resistance (AMR) profiles of *C. jejuni* and *C. coli* isolates in each farm and age group (C, Calves; H, Heifers; and LC, Lactating cows) throughout the sampling period. (**B**) Pie-charts illustrating the relative frequencies of each AMR profile for each *Campylobacter* species. Phenotypic profiles were colour-coded according to legend and antimicrobials were abbreviated as follows: erythromycin (ERY), ciprofloxacin (CIP), nalidixic acid (NAL), streptomycin (STR) and tetracycline (TET).

A total of 170 *C. jejuni* and 37 *C. coli* isolates from faeces (up to 2 isolates per sampling date, age group, and species in each farm) were tested for antimicrobial susceptibility. Overall, resistance rates were higher in calves compared to heifers and lactating cows (χ2 = 6.46, *p* = 0.011) (Supplementary Fig. S1); in fact, all isolates from calves were resistant to at least one antimicrobial class. Resistance was more widespread in *C. coli* compared to *C. jejuni* (χ2 = 7.22, *p* = 0.007). Only 1 *C. coli* isolate (2.7%, 1/37) was susceptible to all antimicrobials tested compared with 25 pansusceptible *C. jejuni* (14.7%, 25/170) (Fig. 1). Among the 181 isolates that were resistant to at least one antimicrobial, 155 (144 *C. jejuni* and 11 *C. coli*) were resistant to both CIP and NAL. Thus, resistance to FQ was very high in *C. jejuni* compared to *C. coli* (84.7% *vs.* 29.7%; OR_adj_ = 15.21 (6.79–34.09), *p* < 0.001) and decreased with age (χ2 = 6.59, p = 0.010); resistance to TET was slightly lower (70.0%, 119/170), and showed a similar age trend (χ2 = 12.12, *p* = 0.002). Resistance to STR was low in *C. jejuni* (8.2%, 14/170) but extremely high in *C. coli* (97.3%, 36/37), levels differing significantly between both species (OR_adj_ = 416.0 (53.55–3243.82), *p* < 0.001). Only in F4 was resistance to STR widespread among *C. jejuni* isolates (5 of the 6 resistant *C. jejuni* isolates). Resistance to ERY was only detected in 3 *C. coli* isolates from two pooled samples collected from calves in F5 during two consecutive samplings. All 207 isolates were susceptible to GEN.

The different profiles of microbiological resistance resulting from the combination of resistance to antimicrobial agents with MICs above the ECOFF and their distribution within each farm are represented in Fig. 1. Between 2 and 4 different profiles of resistance were observed in each farm along the study, diversity being higher in lactating cows and heifers compared to calves, and in F5 and F4 compared to the other farms. Two multidrug resistance (MDR) profiles (three or more classes of antimicrobial agents) were identified, i.e., CIP-NAL-STR-TET, present in *C. jejuni* isolates recovered from all farms except F2 and widespread in *C. coli* from F4, and ERY-CIP-NAL-STR-TET, only detected in *C. coli* recovered from calves in F5. The profile CIP-NAL-TET predominated in F1, F2 and F3. In F4, all but one resistant isolate were resistant to STR alone or in combination with TET and CIP/NAL. In this farm, the predominant profile of resistance in *C. jejuni* (5/6) was CIP-NAL-STR-TET while in *C. coli* 3 different profiles were identified. In F5, despite the low number of isolates recovered (only 5 samplings), diversity was the highest and included 4 *C. jejuni* profiles and the MDR *C. coli* profile ERY-CIP-NAL-STR-TET in calves.

### WGS output and assembly results

A selection of 56 isolates from F1 and F4 (one per *Campylobacter* species, age group, and sampling time) was analysed by WGS to thoroughly compare the genomic profile of the strains circulating in these farms. ONT sequencing of these isolates provided a median of 23,393 reads per sample (IQR = 19,502–29,183) in a median of 302 Mb per sample (IQR = 280–314 Mb) corresponding to a median coverage of 157X (Q1–Q3 = 143–165 X) (Supplementary Dataset S1). Upon assembly, 53 isolates resulted in circularized chromosomes. In all cases, the chromosome size of the assembled draft genome corresponded to the expected size of *C. jejuni* (median = 1,740,992 bp, IQR = 1,690,866–1,743,662 bp) or *C. coli* (median = 1,661,122 bp, IQR = 1,660,938–1,681,616 bp) and G + C content (median = 30.43% and 31.49%, respectively).

Plasflow identified 14 plasmidic contigs and another 12 were proved compatible as plasmids after querying them with blastn. These twenty-six contigs corresponded to complete circular plasmids, 21 in *C. jejuni* and 5 in *C. coli*, that showed high degree of identity with several plasmids from GenBank (Supplementary Dataset S2). ProgressiveMAUVE multiple alignments identified 4 different plasmid structures with a high degree of synteny, and two of them carrying ARGs. Thus, ARG harbouring plasmids in *C. jejuni* (18 isolates from F1 and 1 isolate from F4) had an average size of *ca.* 44,650 bp and were highly similar among themselves. Specifically, a similar Tet-plasmid was found in *C. jejuni* of ST-48 and ST-19 types circulating in F1 whereas the plasmid in ST-45 (C0944) was slightly smaller (43,961 bp) but highly homologous. A slightly larger plasmid (45,150 bp) was found in a *C. jejuni* isolate (C0775) recovered from F4 (Supplementary Fig. S2A). The only resistance gene present in *C. jejuni* plasmids was *tet*(O). The ARG-encoding plasmids in *C. coli* (3 isolates from F4), sized *ca.* 47,150 bp, were identical among themselves, and in addition to the *tet*(O) gene they harboured an aminoglycoside cluster (Δ*ant(6)-Ia*–*sat4*–*aph(3')-IIIa*) (Supplementary Fig. S2B). Plasmids without ARGs were present in 2 *C. jejuni* isolates (sized *ca.* 4,365 bp) and 2 *C. coli* isolates (sized *ca.* 26,700 bp), all recovered from F4.

### Distribution of genetic determinants of resistance in F1 and F4: both farms differed regarding their diversity and location

**Figure 2:**
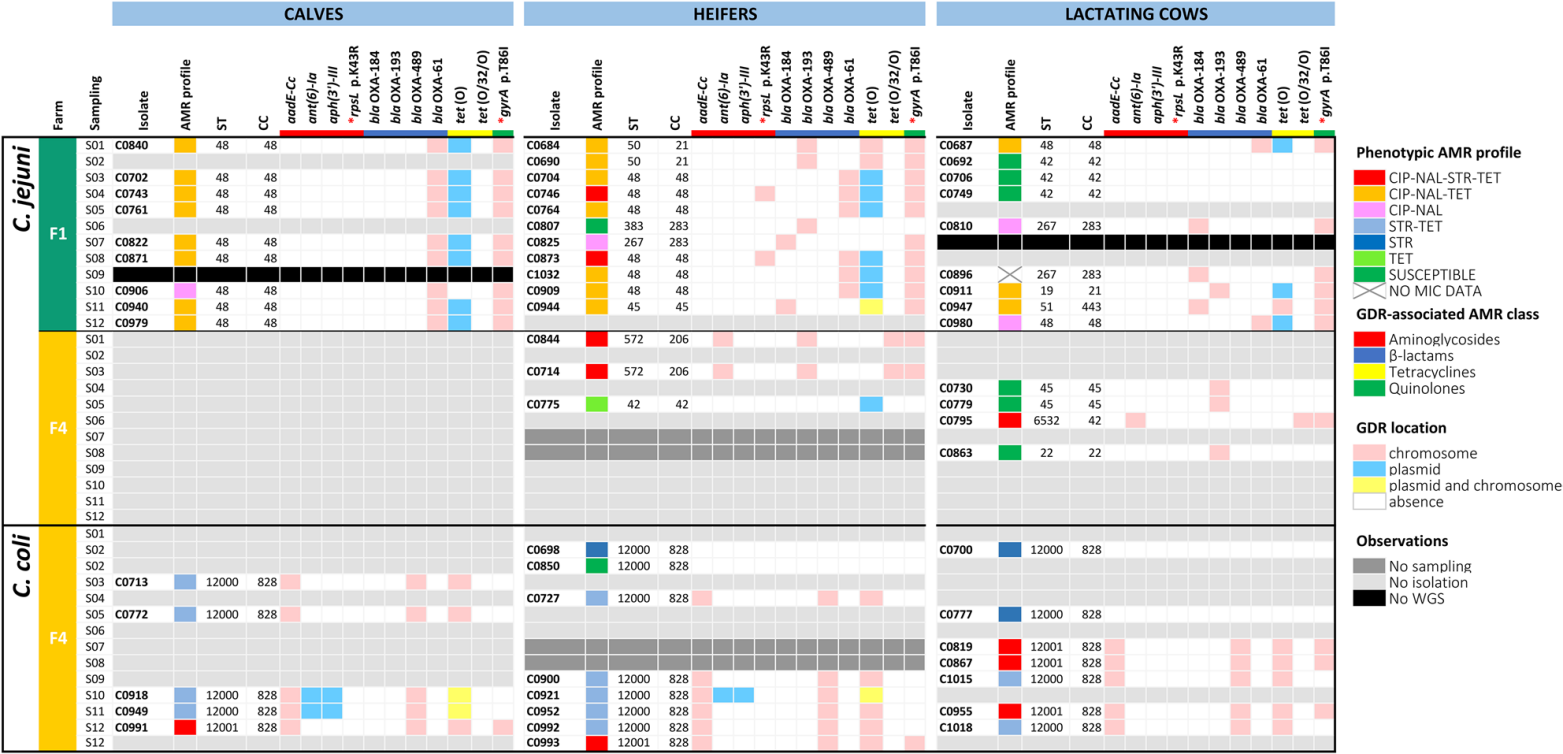
Distribution of genetic determinants of resistance (GDR) of *C. jejuni* and *C. coli* isolates in farm F1 and F4 by age group and sampling point (S01-S12) as detected by WGS. Cells are colour-coded to indicate presence/absence of each GDR and its genomic location. GDRs are sorted according to the class of the antimicrobial resistance they encode. Phenotypic antimicrobial resistance (AMR) profiles are colour-coded according to legend and antimicrobials were abbreviated as follows: erythromycin (ERY), ciprofloxacin (CIP), nalidixic acid (NAL), streptomycin (STR) and tetracycline (TET). Additional metadata such as MLST were provided (ST, sequence type; CC, clonal complex).

Screening for genetic determinants of resistance (GDRs) identified 7 acquired ARGs and point mutations in two other genes associated with resistance to antimicrobials representing 4 different classes (Fig. 2). The combination of GDRs detected in each isolate resulted in 13 different genotypic profiles of resistance (Supplementary Dataset S3). There was an overall very good concordance between susceptible pheno- and genotypes; the only discrepancies were 3 *C. coli* from F4 (C0698, C0700, and C0777) that were phenotypically resistant to STR (MIC = 8 mg/L) but did not carry any GDR associated with STR resistance, and one *C. jejuni* from F1 (C0980) that carried the *tet*(O) gene in a plasmid but was susceptible to TET (MIC ≤ 0.5 mg/L). Resistance to β-lactams was not phenotypically tested but different *bla*_OXA_ genes were identified in 47 isolates; 5 *C. jejuni* isolates from F1 carried the *bla*_OXA-184_ gene and 42 isolates carried different *bla*_OXA-61_-like gene alleles (*bla*_OXA-61_ and *bla*_OXA-193_ in 17 and 9 *C. jejuni*, respectively, and *bla*_OXA-489_ in 16 *C. coli*). In both farms, resistance to FQ was always associated with a SNP mutation (T86I) in the *gyrA* gene in *C. jejuni* and *C. coli*. Tetracycline resistance in *C. jejuni* recovered from F1 was always associated with the *tet*(O) gene, generally plasmid-encoded (n = 17), but also chromosomally-encoded (n = 3) or both (n = 1). In F4, TET-resistant *C. jejuni* carried the *tet*(O/32/O) gene in the chromosome (n = 3) or the *tet*(O) gene in a plasmid (n = 1), while in *C. coli,* TET-resistance was associated with a chromosomally-encoded *tet*(O) gene (n = 16), with 3 isolates also carrying a second copy of the gene in a plasmid. In *C. jejuni*, STR resistance was sporadic and associated with an SNP mutation in *rpsL* (K43R) in 2 isolates from F1 and coded by the *ant(6)-Ia* gene in 3 isolates from F4. Streptomycin resistance in *C. coli* was always mediated by the *aadE*-Cc gene (n = 16). In addition, 3 *C. coli* (1 heifer and 2 calves) harboured an aminoglycoside cluster (*tet*(O)–Δ*ant(6)-Ia*–*sa*t*4*–*aph(3')-IIIa*) in a plasmid. Thus, the vast majority of ARGs were chromosomally-encoded; the only plasmid-encoded ARGs were *tet*(O), present in all plasmids (19 *C. jejuni* and 3 *C. coli*), and *aph(3')-III* (the 3 abovementioned *C. coli*).

### Genomic diversity characterization

MLST typing assigned the 56 isolates to 13 types, including two novel STs identified in *C. coli* from F4 (ST-12000, n = 15 isolates; ST-12001, n = 5) that were the result of new combinations of alleles and only differed between them in the sequence of the *tkt* gene. In F1, 29 *C. jejuni* isolates clustered into 8 MLST STs belonging to 6 clonal complexes (CC). In F4, the 7 *C. jejuni* sequenced were assigned to 5 STs (4 CC) and the 20 *C. coli* to the 2 novel STs (ST12000 and ST12001, both assigned to CC828). Only 2 STs were shared by both farms (Supplementary Fig. S3), i.e., ST-42 (3 isolates in F1 without GDRs and 1 isolate in F4 with *tet*(O) gene in a plasmid) and ST-45 (1 isolate in F1 and 2 in F4 with a different repertoire of ARG in each farm).

When the genomes were searched for the presence/absence of genes coding for virulence factors (VF) in the full dataset of the virulence factor database (VFDB), hits were obtained for 225 VF (all chromosomally-encoded) with different degrees of identity (Supplementary Dataset S4). Three *C. jejuni* isolates from F1 (C0684, C0690, C0911) shared an identical VF profile and were the only isolates that carried genes responsible for LOS synthesis and modification like Cj1135, Cj1136, Cj1137c, Cj1138, *cstIII*, *hldE*, *neuA1*, *neuB1*, *neuC1*, and *wlaN*. The pattern of VF presence/absence was used as a comparative genomic fingerprinting tool to subtype the isolates, showing large genetic diversity among the sequenced isolates. Although the VF profile was occasionally able to discriminate among STs, there was an overall good correlation between MLST types and VF profiles with isolates within the same ST clustering together based on the VF profile.

The genomic relatedness among the strains was elucidated from the phylogenetic trees inferred from the core genome alignments (Supplementary Fig. S4). Although core genome characterization provided more precise subtyping, *C. jejuni* isolates belonging to the same ST and CC clustered together in the core genome SNP-based tree. In *C. coli*, however, ST12000 isolates formed two clusters. It was noteworthy that the same subclustering of ST-12000 *C. coli* isolates was also observed by VF profile analysis. One of the clusters included 3 *C. coli* isolates (C0698, C0700, and C0777) phenotypically resistant to STR (MIC_STR_ = 8 mg/L) but without GDRs and a fully susceptible isolate (C0850); the other cluster included 11 isolates with the same phenotypic AMR profile and chromosomal ARG content.

### ARG transmission dynamics

Identical strains (same MLST, GDR, VF, and core genome SNP-based clustering) were isolated in animals from different age groups within each farm, and certain genomic subtypes predominated in each farm (Fig. 2 and Supplementary Dataset S3). In F1, *C. jejuni* ST-48 was isolated throughout the study in the three age groups; it was the only type found in calves, represented 54.5% (6/11) of the isolates from heifers and was only occasionally found in lactating cows (2/9, 22.2%). All 17 ST-48 isolates were resistant to FQ as a result of a C257T SNP-mutation in the *gyrA* gene, all except one (C0906, calves, S10) harboured a *tet*(O) gene-encoding plasmid, and 2 other isolates (heifers, S04 and S08) differed by being STR-resistant due to an SNP-mutation in the *rpsL* gene. In heifers, 5 other isolates with 4 different genomic profiles were isolated, differing in the presence and/or location (plasmid and chromosomal) of the *tet*(O) gene. In lactating cows, genomic diversity was larger (9 isolates, 5 profiles). Isolates of ST-267 with identical ARG and VF profiles were obtained from lactating cows (S06 & S09) and heifers (S07).

In F4, *C. jejuni* was only sporadically isolated from lactating cows and heifers, each age group hosting different genomic subtypes. Conversely, *C. coli* was more widespread and included 2 MLST types, both detected in all age groups. However, the most prevalent type, ST-12000, predominated in calves and heifers and was detected throughout the study. ST-12001, associated with an MDR profile (CIP-NAL-STR-TET), was first detected in lactating cows during the second half of the study, and was only detected in calves and heifers at the last sampling.

## Discussion

This longitudinal study designed to monitor the occurrence and within-farm transmission dynamics of AMR in *C. jejuni* and *C. coli* in dairy cattle showed a more widespread distribution of *C. jejuni* compared to *C. coli* but higher rates of resistance in *C. coli* than in *C. jejuni*, as described before^6,12,18^. When considering the different age groups analysed, resistance was higher in isolates recovered from calves compared to those obtained from heifers and lactating cows. In fact, all isolates from calves showed resistance to at least one antimicrobial class, likely reflecting age-related differences in management practices and antimicrobial consumption associated to the pathologies typically found in calves (mainly respiratory and digestive disorders). However, the diversity of phenotypic resistance profiles also varied between farms. Therefore, to investigate the molecular mechanisms of resistance and thoroughly compare the relationship of the circulating strains, we performed long-read WGS using ONT on a selection of 56 isolates from two farms: F4, which showed the largest diversity in *Campylobacter* species and AMR phenotypic profiles, and F1, where AMR profile diversity was lower but *C. jejuni* isolation frequency was the highest.

Overall, 7 acquired resistance genes (*aadE*-Cc, *ant(6)-Ia*, *aph(3')-III*, *bla*_OXA-184_, *bla*_OXA-61-like_, *tet*(O) and *tet*(O/32/O)) and SNP mutations in 2 other genes (*gyrA* and *rpsL*) were detected. *bla*_OXA_ was the most widespread gene, but β-lactams were not included in the broth microdilution panel since this class of antimicrobials is not recommended for treating campylobacteriosis. Besides, the G57T transversion upstream of the promoter region of the *bla*_OXA-61-like_ gene that allows the expression of the β-lactamase^19^ was not analysed here. A previous study carried out in the Basque Country showed 38.6% of resistance to ampicillin and 42.9% of the isolates carrying the *bla*_OXA-61-like_ gene had the G57T transversion at the promoter region^16^. For the remaining antimicrobials, there was a very good agreement between phenotypic resistance and the presence of the associated ARGs and/or chromosomal point mutations, with only 4 isolates showing presumptive discrepancies. These included 3 *C. coli* isolates which were most likely misclassified as phenotypically resistant to STR based on the observed MIC (MIC_STR_ = 8 mg/L, 1 dilution above the ECCOF value). These isolates did not carry any associated GDR and shared other genetic features (i.e., ST, core genome SNP-based clustering, VF profile, and absence of GDRs) among themselves and with a fully susceptible isolate, suggesting that the observed MIC for STR was the result of the variance associated to the inherent error for MIC methods (± 1 log_2_ dilution). On the other hand, *C. jejuni* isolate C0980 was susceptible to TET (MIC < 0.5 mg/L) despite carrying the *tet*(O) gene in a plasmid. The presence of the *tet*(O) gene in phenotypically susceptible *Campylobacter* has been reported before^20,21^. Guernier-Cambert *et al.*^21^ reported a P76L substitution to render a non-functional plasmid-borne *tet*(O) gene. Here, after sequencing isolate C0980 twice and performing an in-depth sequence analysis of the *tet*(O) gene (data not shown) we found in both cases a deletion in nucleotide position 1078 that would produce a frameshift and result in a non-functional gene thus explaining the phenotypic susceptibility to TET.

Fully functional TET-resistance encoding genes were found in all 40 phenotypically resistant isolates, with the *tet*(O) gene widespread in both species and the mosaic gene *tet*(O/32/O) restricted to 3 *C. jejuni*. The *tet*(O) gene was chromosomally encoded in *C. coli*, whereas in the majority of *C. jejuni* (19/22) it was located in pTet plasmids (Type 1) that also included several Type IV secretion system (T4SS) genes described to form the core genome of Tet-plasmids in *Campylobacter*^22^. The transfer of plasmid-encoded antibiotic resistance in *Campylobacter* has been frequently reported and has been regarded as the main mechanism of TET resistance in *C. jejuni*^23,24^. The presence of the *tet*(O) gene in more than one copy (one in the chromosome and another one in a plasmid) in 1 *C. jejuni* and 3 *C. coli*, was possibly the result of the acquisition of a Tet-plasmid by an isolate that already carried the gene in the chromosome. The mosaic *tet*(O/32/O) gene is not as widespread in *Campylobacter* as *tet*(O) but interestingly it was found in the chromosome of *C. jejuni* isolates of MLST types already reported to chromosomally harbour *tet*(O/32/O), i.e., ST-6532 and CC206 recovered from cattle and sheep^16^. The presence of the *tet*(O) gene on either plasmids or the chromosome suggests acquisition events occurring either via conjugation or by natural transformation^8,25^ as a result of the constant flow of resistance genes between the bacteria present in the farm environment and the cattle gut microbiome.

MLST types belonging to clonal complexes considered as host-specific for cattle (e.g*.* CC42 and CC22) were detected, but most *C. jejuni* isolates were assigned to host-generalist clonal complexes (CC21; CC45; CC48; CC206), genotypes infectious to humans that are frequently recovered from multiple host species^26,27^. Among these were the 3 *C. jejuni* isolated from lactating cows and heifers in F1 that carried several genes responsible for LOS synthesis and modification. These included *wlaN*, which mediates phase-variation of LOS epitopes responsible for autoimmunity and associated with Guillain–Barré syndrome^28^, *cstIII* (sialyltransferase), responsible for the addition of sialic acid to LOS type C, and the N-acetylneuraminate biosynthesis genes (*neuABC*), as well as other putative outer-core sugar transferases of the LOS such as Cj1135, Cj1136, Cj1137c, and Cj1138. The 3 isolates harbouring these genes were classified into CC21, in agreement with previous descriptions of class C LOS in isolates of this clonal complex originating from humans and poultry^29^.

Core genome analysis, MLST types (ST and CC) and the repertoire of GDRs and VFs were used to characterize and compare isolates recovered from the two farms over the 16-month study period. The persistence of certain genotypes was observed throughout the study while other strains were only sporadically detected. In F1, whereas ST-48 seemed to be adapted to calves outcompeting all other strains, in heifers and lactating cows it circulated simultaneously with several other strains suggesting that multiple source contamination events were more frequent in older animals than in calves. ST-48 isolates clustered together based on core genome SNP analysis and their VF profile and carried a similar repertoire of GDRs. All but one harboured a nearly identical Tet-plasmid that was also similar to that found in an isolate of a different MLST type (ST-19) recovered from lactating cows at the end of the study (S10). Homology searches using blastn showed a high percentage of identity between these and other plasmids found in GenBank, including those previously found in *C. jejuni* from sheep and cattle from the same region^16^. These findings suggest that these represent ubiquitous pTet *Campylobacter* plasmids known to transfer resistance to tetracycline.

In F4, *C. jejuni* was only sporadically isolated from heifers and lactating cows and no genotypes were shared between both age groups. Conversely, *C. coli* was widespread, but MLST diversity was lower; just 2 STs (ST-12000 and ST-12001) that only differed between them in the *tkt* allele and were closely related (different *glnA* alleles) to other widespread STs like ST-1055 and ST-827, respectively. The lower genetic diversity of *C. coli* of ruminant origin compared to *C. jejuni* or *C. coli* isolated from swine or poultry has been reported previously^30,31^. However, both genotypes were new and never described before, indicating that diversity is greater than reported. All resistant *C. coli* shared a similar repertoire of chromosomally encoded GDRs, and major differences were observed in 3 ST-12000 *C. coli* isolates due to a 47,150 bp plasmid that harboured additional ARGs (*tet*(O)–Δ*ant(6)-Ia*–*sat4*–*aph(3’)-IIIa*). Considering only chromosomally encoded GDRs, the only difference between both STs was the presence of a FQ-resistance SNP mutation (C257T) in the *gyrA* gene of all ST-12001 isolates that was absent in ST-12000 isolates. In F4, the FQ-resistant genotype (ST-12001) was detected during the second half of the study, first in lactating cows (S07, S08, S11) and later (S12) in heifers and calves. In addition to providing FQ-resistance, this SNP in the *gyrA* gene, known to be involved in DNA supercoiling^32^, delivers enhanced fitness during commensal colonization^8^. In this sense, FQ resistance in *C. jejuni* has been associated with an increase in virulence and the ability to form viable biofilms in oxygen-rich environments^9^. This might play a critical role in the persistence of FQ-resistant isolates in the animal reservoirs even in the absence of antibiotic selection pressure thus explaining the increasing trend in FQ resistance in *Campylobacter*. The emergence of the FQ-R genotype (ST-12001) and the plasmid-harbouring ST-12000 isolates occurred in both cases near the end of the study and, therefore, it was not possible to monitor their possible spread and persistence in the farm. Additional samplings would have been needed to see whether the FQ-R genotype turned out to be better adapted and able to out-compete the FQ-S through local clonal expansion and to monitor possible further dissemination of the *C. coli* plasmid to other isolates.

In conclusion, this longitudinal study illustrated the within-farm diversity and transmission dynamics of resistant *Campylobacter* throughout time in dairy cattle farms, enabling the detection of changes in the AMR profiles and the emergence of different genotypes over time. Both persistent and sporadic strains were detected, and examples of processes that led to the emergence of novel genomic types that could spread within the herds were proposed, i.e., acquisition of extracellular genes from other isolates and intracellular mutational events. Several genotypes were simultaneously present within each herd but in some instances certain genotypes seemed to be more adapted and persisted for up to 16 months (e.g*.* ST-48 in F1 and ST-12000 in F4). Long-read whole genome sequencing-based surveillance helped to decipher the complex epidemiology underlying the within-farm dissemination of resistant *Campylobacter*.

## Methods

### Study design and sample collection

Five commercial farms (designated F1, F2, F3, F4, and F5) in the Basque Country (northern Spain) representative of the style of farming in the region were enrolled in the study. All farms were closed production systems where replacement heifers originated from the same farm. Two of the farms (F2 and F4) raised their heifer replacements off-site in two different breeding centres. Farms were located in the three counties of the Basque Country, and the distance between farms ranged from 15 to 25 km for those located within the same county (i.e., F3–F4 and F1–F2, respectively) and up to 160 km (F4–F5). Further details on general information about farm characteristics, management practices, vaccine programs, and antimicrobial drug use were reported elsewhere^33^.

The sampling strategy, planned as a year-long study with monthly samplings, was interrupted by the COVID-19 pandemic and extended from February 2019 to October 2020. Rectal faeces were collected from calves (1–5 months-old), heifers (young cows that have not yet given birth to a calf, 6–22 months-old approx.), and lactating cows (animals in the milking period at sampling) during 12 samplings carried out over 16 months; one of the farms (F5) dropped out after 5 samplings due to operational changes. At each sampling time and farm, five animals were randomly selected within each age group and rectal faeces (minimum of 5 g) were collected with a gloved hand, and faeces of the 5 animals of the same age group were analysed in a single 25 g pool. In farms F2 and F4, heifers were at the breeding centre at several sampling dates (5 samplings in F2 and 2 in F4) and faeces could not be collected. Thus, a total of 760 rectal faecal samples were collected and analysed in 152 pools.

Sample collection was carried out by veterinary practitioners strictly following Spanish ethical guidelines and animal welfare regulations (Real Decreto 53/2013). The collection of this material, being considered a routine veterinary practice, did not require the approval of the Ethics Committee for Animal Experimentation. Informed oral consent was obtained from the farm owners at the time of sample collection. All methods were performed in accordance with the relevant guidelines and regulations and complied with ARRIVE guidelines^34^.

### Isolation and identification of *Campylobacter* species

For the isolation of thermophilic *Campylobacter* spp., 25 g of pooled rectal faecal samples were diluted 1:10 in Preston broth, homogenized, and incubated for 18 ± 2 h at 42 ℃ for enrichment. Suspensions (0.1 mL) were then subcultured onto a Chromogenic-*Campylobacter* Selective Agar (CASA® Agar, Biomerieux) and incubated at 42 ℃ in a microaerobic atmosphere (5% O_2_, 10% CO_2_, 85% N_2_) for 48–72 h. To confirm the presumptive *Campylobacter* and identify the species present, 10 individual colonies were tested using a multiplex real-time PCR targeting *C. jejuni mapA* gene and *C. coli ceuE* gene^35^. When neither *C. jejuni* nor *C. coli* could be identified by colony picking, DNA was extracted from a loopful of bacterial culture in CASA® agar (InstaGene, BioRad, CA, USA) and screened for the presence of *Campylobacter* spp. by a real-time PCR that amplifies the 16S rRNA gene of *Campylobacter* genus^36^. A maximum of 2 *C. jejuni* and 2 *C. coli* colonies per sample were stored for further characterization.

### Antimicrobial susceptibility test determination by broth microdilution

When available, 2 isolates per plate and *Campylobacter* species (i.e., *C. jejuni* and *C. coli*) were selected and tested to assess antimicrobial susceptibility. Minimum inhibitory concentrations (MIC) were determined by broth microdilution using Sensititre® EUCAMP2 Susceptibility Plates (ThermoFisher Scientific, Waltham, MA, USA) containing two-fold serial dilutions of six antimicrobial agents: gentamicin (GEN), streptomycin (STR), tetracycline (TET), ciprofloxacin (CIP), nalidixic acid (NAL) and erythromycin (ERY). Antimicrobials and serial dilution ranges were selected following recommendations by the Commission Implementing Decision 2013/652/EU. MIC results were interpreted using epidemiological cut-off (ECOFF) values as developed by the European Committee for Antimicrobial Susceptibility Testing (EUCAST, http://www.eucast.org) to define microbiological resistance to the antimicrobial in question, that is, to discriminate those microorganisms with and without acquired resistance mechanisms (mutant and wild type, respectively).

### Whole-genome sequencing (WGS) and bioinformatic analyses

Fifty-six isolates from F1 (29 *C. jejuni*) and F4 (7 *C. jejuni* and 20 *C. coli*) were selected based on their sampling time and age group source and subjected to long-reads (Oxford Nanopore Technologies, ONT) WGS. Genomic DNA was extracted from pure cultures using NZY Microbial gDNA Isolation kit (NZYtech) and a library was prepared using the ONT Ligation Sequencing Kit (SQK-LSK109). Native barcoding genomic DNA kits (EXP-NBD104 and EXP-NBD114) were used for sample multiplexing and libraries were run in FLO-MIN106 (R9.4.1) or FLO-MIN111 (R10.3) flow cells on a MinION Mk1C device (ONT). The output files generated by ONT sequencing were base-called in high accuracy mode (HAC) and quality-filtered using Guppy v.5.0 (Qscore > 8). Reads were adapter-trimmed with Porechop v.0.2.4 with the default parameters^37^ (Wick 2017) and filtered by length and quality using Filtlong v.0.2.0 (https://github.com/rrwick/Filtlong) by discarding short reads (< 1000 bp) and keeping the best 90% of the remaining reads for further analyses (–min_length 1000 –keep_percent 90). Then, the resulting fastq reads were de novo assembled using Unicycler^38^. MLST profiles were determined from unassembled long-reads using Krocus^39^. New combinations of existing alleles along with representative isolate data were submitted to the *Campylobacter* MLST database pubMLST for new ST definition^40^. Genomes were processed to predict plasmid- and chromosome-derived contigs using PlasFlow (v.1.1) (Krawczyk et al.^41^), and small circular contigs were queried against blastn database (https://blast.ncbi.nlm.nih.gov/Blast.cgi) using default parameters and the lowest E-value was considered the best hit. BLASTn v.2.12.0+^42^ and ABRicate v.1.0.1 (T. Seemann, https://github.com/tseemann/abricate) were used to screen the draft genomes against ResFinder^43^ for the detection of acquired antimicrobial resistance genes and against VFDB_setB (full dataset) for virulence factors. Chromosomal point mutations associated with AMR were investigated by screening unassembled reads against the PointFinder database^44^ using Resfinder v.4.1.0^45^. Databases used were all updated on 11/05/2022. ﻿Resfinder hits were filtered at 90% coverage and identity. Virulence genes were filtered at 90% coverage and 60% identity, and the pattern of presence/absence of these genes was used as a typing scheme for comparative genomic fingerprinting, supported with a dendrogram. The hierarchical clustering analysis for the dendrogram was performed with the unweighted pair-group method with arithmetic mean (UPGMA) based on the Jaccard distance matrix, using the function hclust (v.3.6.1) of the R statistical package v.3.6.3. Plasmid alignments were performed using MAUVE in progressive mode^46^ in Geneious Prime v. 2020.2.4 (https://www.geneious.com) software and GDR arrangements were graphically represented using SnapGene v.5.2.4 (http://www.snapgene.com/). Parsnp v1.7.4^47^ along with the implemented RaxML v.8.2.12^48^ was used to perform core genome analyses and construct phylogenetic trees based on the chromosomal genomes to identify structural and point variations (SNPs), with defaults parameters and specifying -r ! parameter to randomly select the reference from the set of genomes analysed. The resulting trees were visualised with iTOL^49^.

### Statistical analysis

Differences in AMR prevalence between the different *Campylobacter* species, age groups, and/or farms were evaluated with multivariate logistic regressions. Adjusted odds ratios (OR_adj_) were used as the measure of association between positivity and the explanatory variables and were expressed with their confidence interval at 95% (95% CI). Differences were considered statistically significant if *p* < 0.05. Analyses were conducted using statistical software Stata/IC version 16.1 (StataCorp LP, College Station, TX, USA).

## Supplementary Information

Supplementary Information 1.

Supplementary Information 2.

## Data Availability

Raw sequencing data of the 56 strains analysed in this study are available at NCBI Sequence Read Archive (SRA) database under accession numbers as detailed in Supplementary Dataset S5, associated with the BioProject PRJNA 932719.
