# Supplementary material for: Monitoring within-farm transmission dynamics of antimicrobial-resistant Campylobacter in dairy cattle using broth microdilution and long-read whole genome sequencing
Source: Sci Rep. 2023 Aug 2;13:12529. doi: 10.1038/s41598-023-39588-3 (PMC10397349; doi:10.1038/s41598-023-39588-3)

Supplementary Information file

Monitoring within-farm transmission dynamics of antimicrobial-resistant *Campylobacter* in dairy cattle using broth microdilution and long-read whole genome sequencing

Medelin Ocejo[1], Beatriz Oporto[1], José Luis Lavín[2] and Ana Hurtado[1,*]

[1] Animal Health Department, NEIKER – Basque Institute for Agricultural Research and Development, Basque Research and Technology Alliance (BRTA), Bizkaia Science and Technology Park 812L, 48160 Derio, Bizkaia, Spain.

[2] Applied Mathematics Department, NEIKER – Basque Institute for Agricultural Research and Development, Basque Research and Technology Alliance (BRTA), Bizkaia Science and Technology Park 812L, 48160 Derio, Bizkaia, Spain.

*ahurtado@neiker.eus

Figure S1. Percentage of isolates resistant to each antimicrobial tested as determined by the broth microdilution. Antimicrobials were abbreviated as follows: gentamycin (GEN), streptomycin (STR), tetracycline (TET), ciprofloxacin/nalidixic acid (CIP/NAL) and erythromycin (ERY).

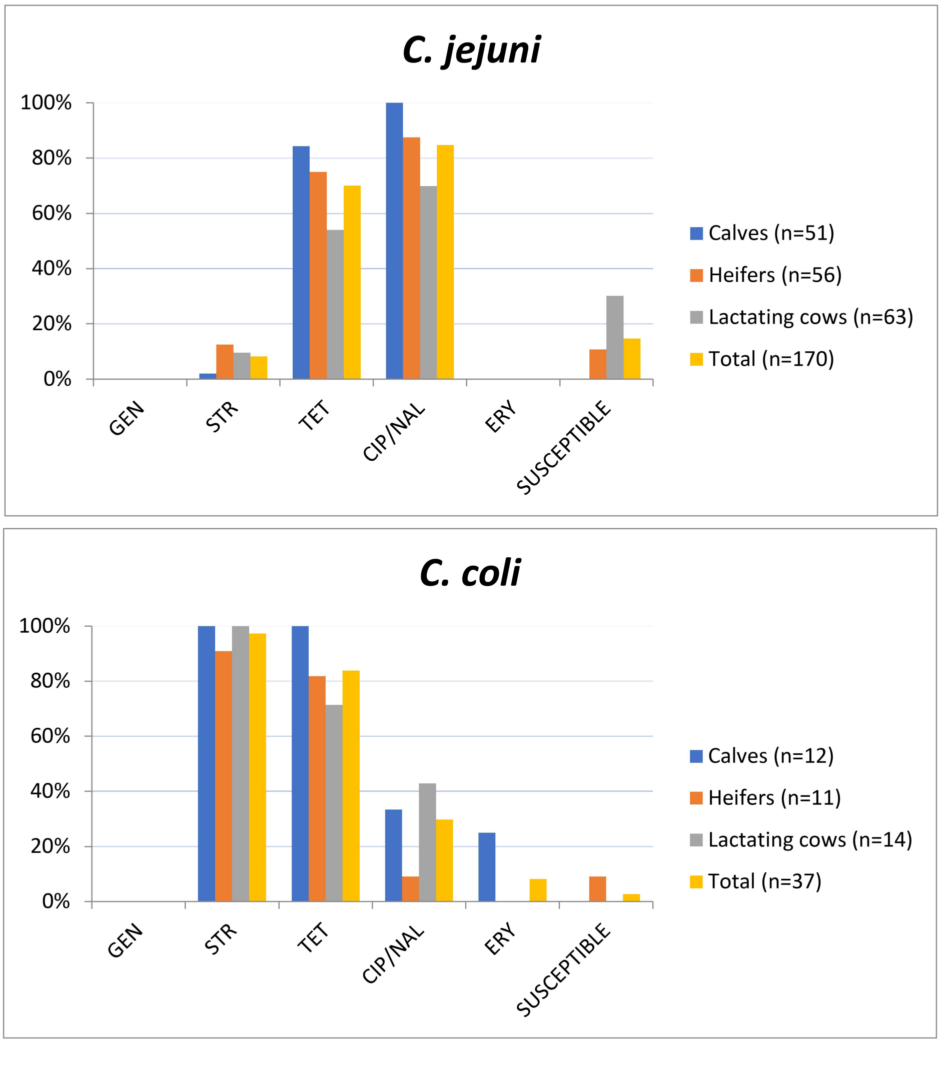

Figure S2. Structural comparison of *C. jejuni* (A) and *C. coli* (B) ARG-harboring plasmids using progressiveMAUVE multiple genome alignment. The pink colored region is a locally collinear block (LCB) and the presence of only one block indicates high homology among plasmids and similar gene arrangements. The positions of the antimicrobial resistance genes (ARGs) are indicated in colored rectangles (*tet*(O) in blue and the aminoglycoside cluster in yellow) and represented below.

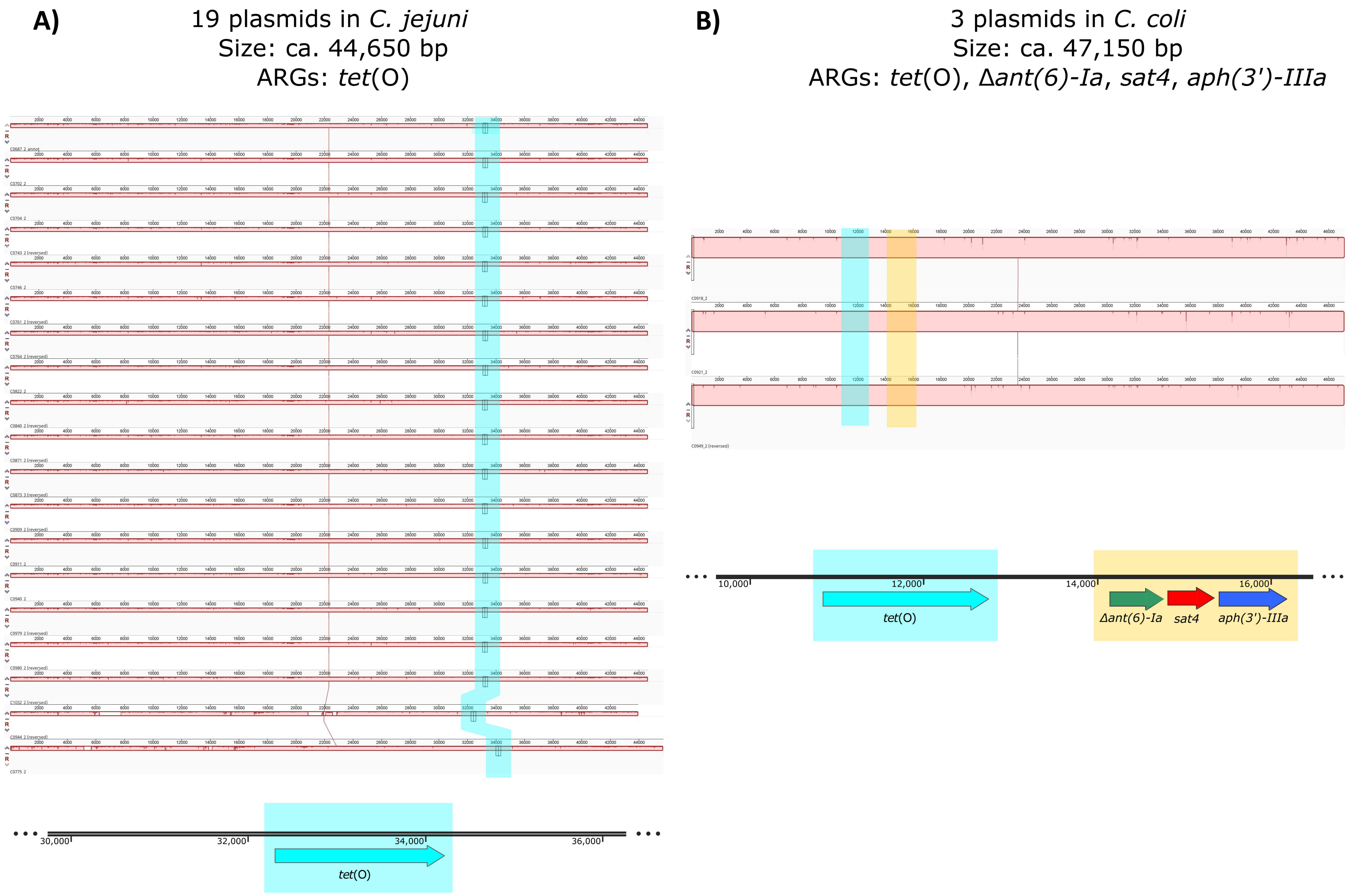

Figure S3. Venn diagrams showing the distribution of the different MLST types assigned to *C. jejuni* and *C. coli* isolates in farms F1 and F4. The number of isolates for each ST is provided between parentheses. For STs present in both farms the number of isolates from F1 are indicated first followed by those from F4.

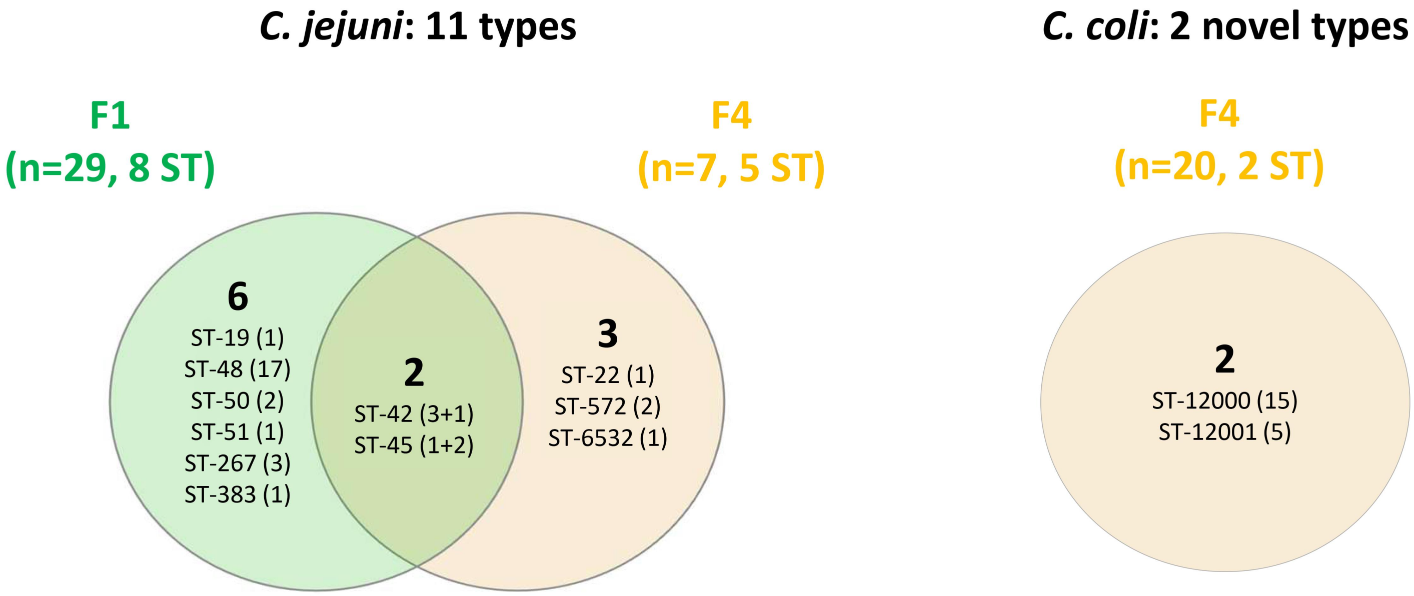

Figure S4. SNP-based core genome phylogeny of *C. jejuni* (A) and *C. coli* (B) isolates of the study. The tree represents the genetic relationships among isolates based on their core genomes. The phylogenetic tree was constructed using Parsnp and RaxML, and corresponding metadata such as farm, age group, sampling time, MLST types and phenotypic and genotypic antimicrobial resistance profiles are indicated for each isolate. Genetic determinants of resistance are color-coded according to genomic location: black for chromosomal, green for plasmidic, and red when there are two copies, one in the chromosome and another in the plasmid.

A)

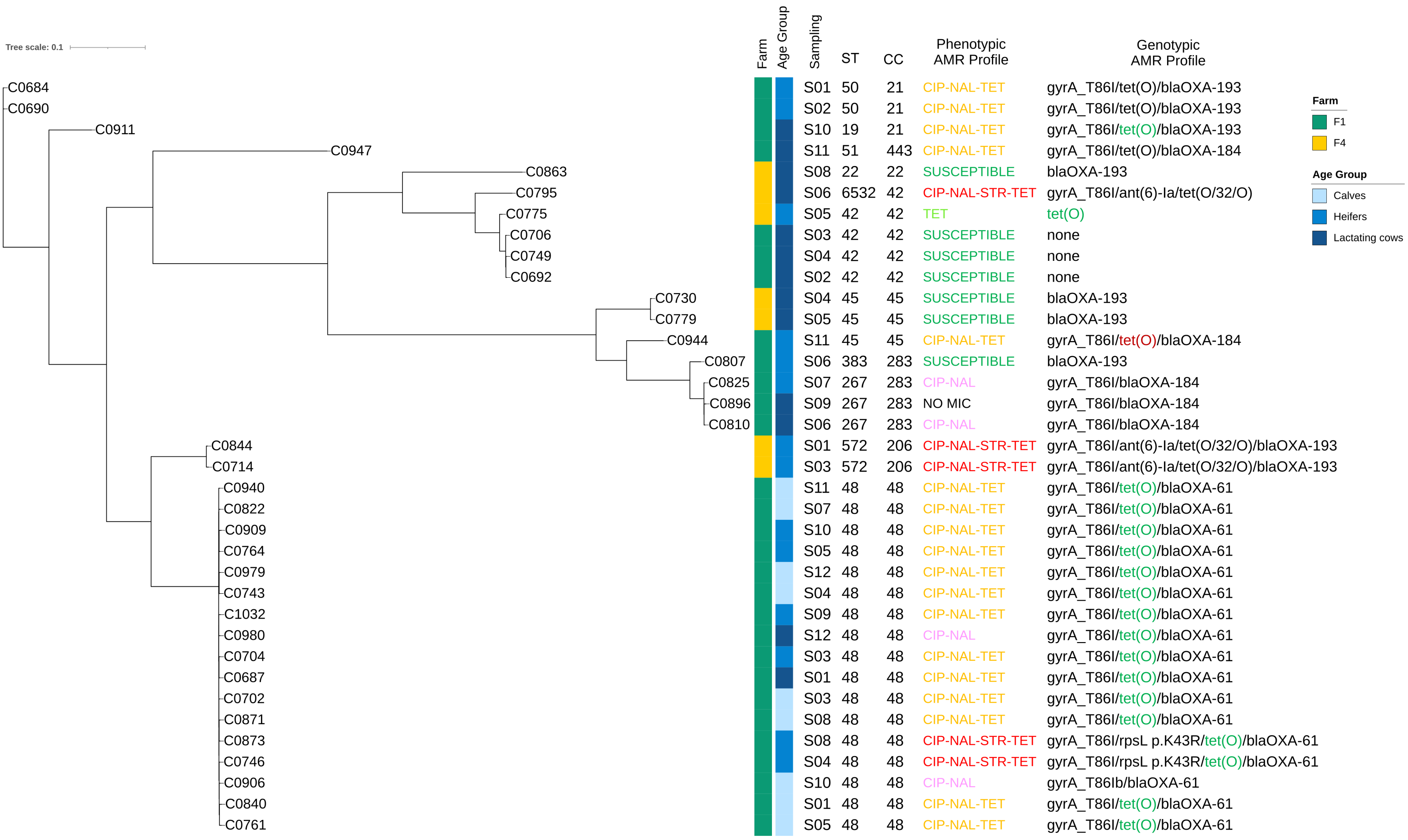

B)

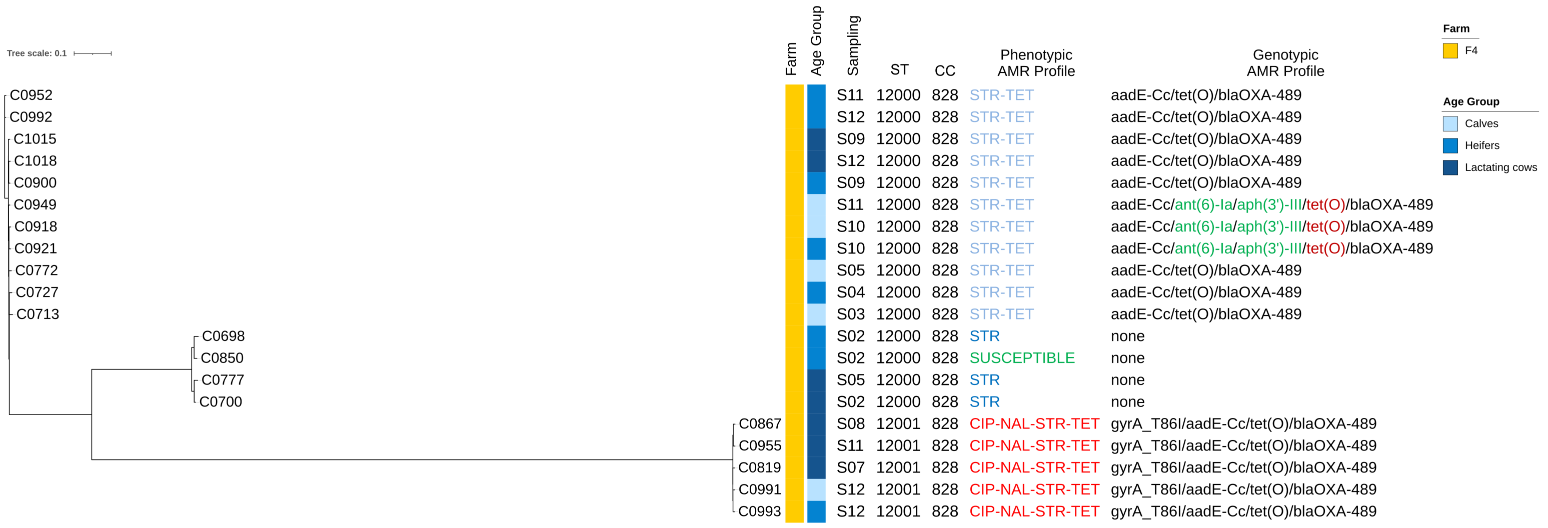

Supplement: Supplementary file 1 — Supplementary Information 1. [file 41598_2023_39588_MOESM1_ESM.pdf]
